# Association of the *TGFB1* Gene Polymorphisms with Pain Symptoms and the Effectiveness of Platelet-Rich Plasma in the Treatment of Lateral Elbow Tendinopathy: A Prospective Cohort Study †

**DOI:** 10.3390/ijms26062431

**Published:** 2025-03-08

**Authors:** Alicja Jarosz, Justyna Wrona, Anna Balcerzyk-Matić, Karol Szyluk, Tomasz Nowak, Tomasz Iwanicki, Joanna Iwanicka, Marcin Kalita, Wojciech Kania, Katarzyna Gawron, Paweł Niemiec

**Affiliations:** 1 Department of Biochemistry and Medical Genetics, Faculty of Health Sciences in Katowice, Medical University of Silesia in Katowice, Medykow 18 Str., 40-752 Katowice, Poland; alicja.jarosz@sum.edu.pl (A.J.); justyna.wrona@sum.edu.pl (J.W.); abalcerzyk@sum.edu.pl (A.B.-M.); tnowak@sum.edu.pl (T.N.); tiwanicki@sum.edu.pl (T.I.); jiwanicka@sum.edu.pl (J.I.); 2 District Hospital of Orthopaedics and Trauma Surgery, Bytomska 62 Str., 41-940 Piekary Slaskie, Poland; kszyluk@o2.pl (K.S.); marcin.kalita1991@gmail.com (M.K.); 3 Department of Physiotherapy, Faculty of Health Sciences in Katowice, Medical University of Silesia in Katowice, Medykow 12 Str., 40-752 Katowice, Poland; 4 Department of Trauma and Orthopedic Surgery, Multidisciplinary Hospital in Jaworzno, Chelmonskiego 28 Str., 43-600 Jaworzno, Poland; wojtekkania@poczta.onet.pl; 5 Department of Molecular Biology and Genetics, Faculty of Medical Sciences in Katowice, Medical University of Silesia, Medykow 18, 40-752 Katowice, Poland; kgawron@sum.edu.pl

**Keywords:** *TGFB1*, TGF-β1, transforming growth factor beta 1, PRP, platelet-rich plasma, lateral elbow tendinopathy

## Abstract

The regenerative properties of platelet-rich plasma (PRP) result from the high concentration of growth factors, including transforming growth factor beta 1 (TGF-β1). Nevertheless, this form of therapy may not always be effective due to the variability in genetic factors. In this study, the association of *TGFB1* gene polymorphisms with the effectiveness of lateral elbow tendinopathy (LET) treatment with PRP was investigated. The effectiveness of therapy was assessed using minimal clinically important difference (MCID) and patient-reported outcome measures (PROM), specifically visual analog scale (VAS), quick version of disabilities of the arm, shoulder, and hand score (QDASH), and patient-rated tennis elbow evaluation (PRTEE) for two years (in weeks 2, 4, 8, 12, 24, 52, and 104). The most effective therapy was noticed in CC rs2278422 genotype carriers, whereas carriers of AA, CC, and CC genotypes (rs12461895, rs4803455, rs2241717) showed more severe pain before therapy. Moreover, the analyses revealed an association of studied polymorphisms with such parameters of blood morphology as eosinophils (EOS), neutrophils (NEU), and monocytes (MONO). In conclusion, genotyping of rs2278422 variant may be a valuable diagnostic method for patient selection for PRP therapy, while genotyping of rs12461895, rs4803455, and rs2241717 polymorphisms may be used for prediction of increased risk of pain sensation.

## 1. Introduction

As reported, there are some interindividual differences in the effectiveness of platelet-rich plasma (PRP) therapy [1]. These differences may result from many factors, including growth factors’ concentration [2], sex [3], age [4], and genetic variability [5]. Transforming growth factor beta 1 gene (*TGFB1*) seems to be a suitable candidate for analysis of PRP effectiveness in tendinopathy due to its association with wound healing [6], tendon development [7], and pain sensation [8] and its presence in PRP [9]. The TGF-β1 exerts pleiotropic action on cells depending on the nature of signaling and cell type, e.g., it induces cytostasis, apoptosis, dormancy, and autophagy [10]. Although initially thought to stimulate cell proliferation, TGF-β1 is a bifunctional regulator that inhibits or stimulates cell division [6] and significantly accelerates wound healing [6,11,12]. Expression and activation of this cytokine are induced in response to injury, progressing outward from the site of contusion [6]. Platelets store large amounts of TGF-β1 that are released at the site of the injury and inflammation to attract monocytes and fibroblasts for local tissue repair [6,11]. It has also been shown that TGF-β1 stimulates the expression of extracellular matrix (ECM) proteins, such as fibronectin, collagen types I and III, and vascular endothelial growth factor (VEGF) [6,12,13], as well as inhibits degradation of ECM. The latter is observed due to repression of the metalloproteinases (MMP) expression [6,12] and stimulation of tissue inhibitor of metalloproteinases (TIMPs) synthesis [6]. Furthermore, TGF-β1 stimulates angiogenic properties of endothelial progenitor cells, facilitating blood flow to the damaged site [8] and plays a pivotal role in fracture healing [14]. Nevertheless, overexpression of this growth factor may lead to fibrosis, scar formation [11,12,13], and alteration in tissue architecture, which may further affect its physiological function [15]. Therefore, in the early stage of injury, TGF-β1 can reduce inflammation and accelerate wound healing, although, at the late stage, it may lead to tissue scarring [16].

The *TGFB1* is expressed in the tendon and likely plays a role in its development [7]. It has been shown that TGF-β1 inhibits the expression of scleraxis and promotes the expression of tenomodulin [17]. Scleraxis is expressed at an early stage of tendon development, initiates tendon differentiation, and inhibits the maturation of collagen fibers during tenogenic differentiation, while tenomodulin is a marker of mature tendon cells and accelerates tendon development [16]. The TGF-β1 also promotes tendon stem cell proliferation and migration, which is beneficial for the treatment of damaged tendons [18]. Additionally, TGF-β1 stimulates the expression of collagen and can reduce inflammatory response [12], which can stimulate tendon healing.

The TGF-β1 also appears to be involved in pain perception. This cytokine is an important mediator of nociception and has a protective effect against the development of chronic neuropathic pain by inhibiting neuroimmune responses [19]. Studies in mice have shown that TGF-β1 secreted from bone marrow stromal cells inhibits neuropathic pain [20], as well as exhibits antinociceptive properties in a model of lumbar dorsal root ganglia inflammation [21]. In studies on rats, TGF-β1 was observed to prevent the development of pain and inflammation during intervertebral disc degeneration [22]. Studies on humans have shown that in patients with osteoporosis, the level of TGF-β1 decreased with increased pain intensity (both in plasma and central cerebral spinal fluid), suggesting that this growth factor might alleviate pain [8]. However, other studies have shown the opposite results, stating that TGF-β1 has pro-pain properties [21,23,24]. The TGF-β1 contributed to the development of pain sensations in both mouse and rat models of bone cancer [23] and was involved in hyperalgesia in rats with chronic pancreatitis [24]. The reasons for these discrepancies are not clear, although they illustrate the vast array of signaling pathways activated by TGF-β1. It is suspected that the effect of TGF-β1 on pain perception depends on the transforming growth factor beta receptor 1 (TGF-βR1) and the activated signaling pathway [21].

Considering that TGF-β1 exerts pleiotropic effects on cells and tissues, its impact on the effectiveness of tendinopathy treatment with the use of PRP is unknown and, so far, has not been studied. Therefore, in the present study, we analyzed the possible association between four single nucleotide polymorphism (SNPs) of the *TGFB1* gene (namely, rs2278422, rs12461895, rs4803455, and rs2241717), with clinical phenotype of lateral elbow tendinopathy (LET), pain perception, and PRP therapy effectiveness of LET treatment.

## 2. Results

### 2.1. Characteristics of the Studied TGFB1 Gene Polymorphisms

**Figure 1:**
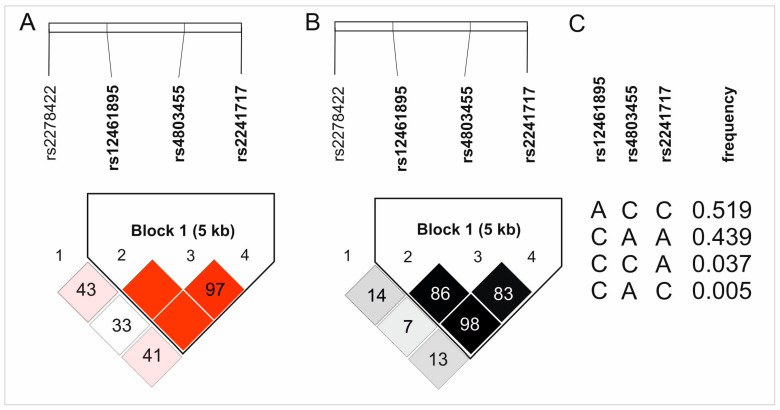
Results of haplotype analyses of the studied *TGFB1* gene polymorphisms: (**A**) D’, (**B**) R^2^, and (**C**) haplotypes frequencies. The color intensity reflects the values of D’ and R^2^.

All studied variants of four analyzed *TGFB1* SNPs are intronic [25] and follow Hardy–Weinberg equilibrium (HWE), except for rs4803455. Interestingly, in the case of rs12461895 and rs2241717, the alleles defined as minor alleles (MAF) in the European population occurred more frequently in our study group. It should be emphasized, however, that these polymorphisms were still consistent with the HWE. The frequencies of the analyzed polymorphisms and the *p*-values for the HWE test are presented in Table 1. Moreover, rs12461895, rs4803455, and rs2241717 SNPs were in strong linkage disequilibrium and formed a haplotype. Figure 1 shows the D’ and R^2^ values from haplotype analyses along with the haplotype frequencies.

### 2.2. TGFB1 Gene Polymorphisms and Blood Morphological Parameters

The studied polymorphisms showed a relationship with blood morphological elements. In the dominate/recessive model, the C allele carriers of rs2278422 had higher levels of neutrophils (NEU); the GG homozygotes presented increased percentage of lymphocytes (LYM), while CC homozygotes increased mean platelet volume (MPV), both in whole blood and PRP. In the case of rs12461895, carriers of the AA genotype showed elevated levels of mean corpuscular hemoglobin concentration (MCHC); CC homozygotes had higher plateletcrit (PCT) and monocyte (MONO) levels, whereas the A allele carriers showed increased eosinophils (EOS) level. Furthermore, CC homozygotes of rs4803455 were characterized by higher platelet distribution width (PDW), AA homozygotes showed increased amounts of red blood cells (RBC), EOS, LYM, and MONO, and carriers of the C allele had raised NEU and white blood cells (WBC) levels. Finally, in the case of rs2241717, CC homozygotes showed elevated levels of PDW and EOS; AA homozygotes showed an increase in platelet (PLT), PCT, and MONO, while C allele carriers presented increased MCHC levels. After the Hochberg correction for multiple testing, the *p*-value was assessed as 0.004. Therefore, the results were still statistically significant only for rs4803455 and NEU concentration, as well as rs2241717 and MONO. The results indicating associations between analyzed SNPs and blood morphological parameters are presented in Table 2.

In the additive model, CC homozygotes of rs2278422 showed increased MPV and NEU concentration, while GG homozygotes had higher LYM levels. AC heterozygotes of rs12461895 were characterized by higher MCHC, while AA homozygotes showed an elevated percentage of EOS. Moreover, AC heterozygotes of rs4803455 presented increased levels of NEU as well as a lower percentage of EOS and LYM. Finally, CC homozygotes of rs2241717 showed an increase in EOS; AA homozygotes had raised MONO concentration, while AC heterozygotes had elevated MCHC. For the additive model, the *p*-value after Hochberg correction was 0.009, leaving a statistically significant association of rs4803455 with NEU and EOS concentration as well as the association of rs2241717 with the MONO level (Supplementary Table S1).

### 2.3. TGFB1 Gene Polymorphisms and PROMs Values

**Figure 2:**
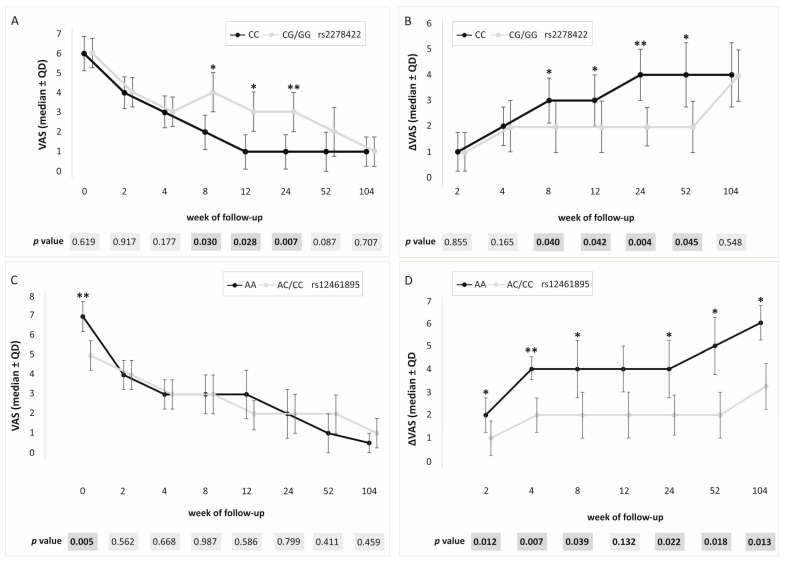
Medians (±QD) of PROM values for carriers of different genotypes. Results for (**A**) rs2278422 VAS, (**B**) rs2278422 ΔVAS, (**C**) rs12461895 VAS, (**D**) rs12461895 ΔVAS. Legend: QD, quartile deviation; PROM, patient-reported outcome measure; VAS, visual analog scale; * significant difference (*p* < 0.050); ** statistically important after Hochberg correction (threshold of significance: *p* ≤ 0.007).

In the dominant/recessive model, the association with patient-reported outcome measures (PROM) values was demonstrated for all analyzed SNPs. We used three different PROM questionnaires: visual analog scale (VAS), quick version of disabilities of the arm, shoulder, and hand score (QDASH), and patient-rated tennis elbow evaluation (PRTEE). For rs2278422, CC genotype carriers had lower VAS (weeks 8–24), QDASH (weeks 12 and 24), and PRTEE (weeks 8–24), as well as higher ΔVAS (weeks 8–52) and ΔQDASH (week 24) values (Figure 2). Moreover, carriers of the C allele presented increased values of ΔQDASH (week 8) and QDASH on day 0 (before PRP injection). In the case of rs12461895, increased values of ΔVAS at almost all follow-up points (with the exception of week 12) and VAS on day 0 were found in AA homozygotes (Figure 2). A higher ΔPRTEE value was also shown for CC homozygotes at week 2, but this result was on the verge of significance (*p* = 0.049). In rs4803455 CC homozygotes, higher values of ΔVAS (weeks 2, 4, 24–104) and VAS (day 0) were observed. Moreover, C allele carriers showed an increased ΔQDASH (week 52) and a decreased PRTEE (week 52). In addition, similar results were obtained in the case of CC rs2241717 genotype carriers, and higher ΔVAS (weeks 2, 4, 24–104) and VAS (day 0) were noted. The *p*-value after correction for multiple testing was set at 0.007. Therefore, statistically significant remaining results for rs2278422 (VAS and ΔVAS at week 24), as well as rs12461895 (ΔVAS at week 4 and VAS at day 0) are shown in Supplementary Tables S2–S5.

**Figure 3:**
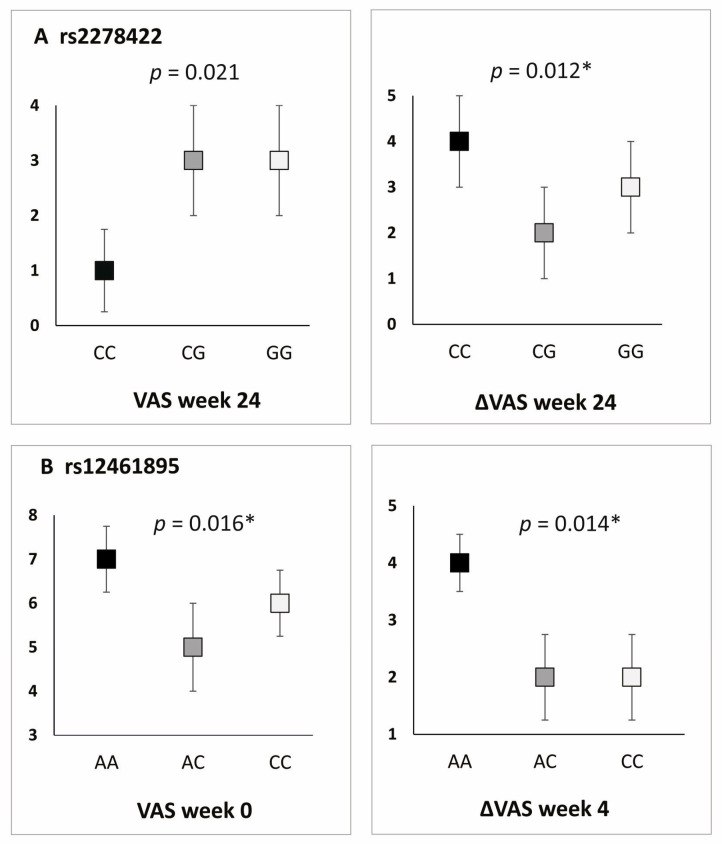
Differences in PROM values for carriers of different *TGFB1* gene genotypes, additive model. Results for (**A**) rs2278422, (**B**) rs12461895. * statistically significant differences after Hochberg correction (threshold of significance: *p* ≤ 0.016).

All studied polymorphic variants showed an association with PROM values in the additive model. In the case of rs2278422, CC homozygotes showed lower values of VAS and PRTEE (both at week 24), whereas, for rs12461895, the AA genotype was associated with increased VAS (day 0) and ΔVAS (weeks 2, 4, and 104) (Figure 3). Similar results were obtained for rs4803455 and rs2241717. CC homozygotes were characterized by higher VAS (day 0) and higher ΔVAS (rs4803455 weeks 2–4, rs2241717 week 4). The threshold of significance for multiple testing was assessed as *p* ≤ 0.016, leaving a statistically significant result for rs2278422 (ΔVAS at week 24) as well as rs12461895 (VAS at day 0 and ΔVAS at week 4) (Supplementary Table S6).

### 2.4. TGFB1 Gene Polymorphisms and Pain Before Therapy

PROM results showed an association of analyzed SNPs with the level of pain before the therapy. Patients with the AA rs12461895, CC rs4803455, and CC rs2241717 genotypes had higher VAS values on day 0 (both in the dominant/recessive and additive models), while C allele carriers of rs2278422 had higher QDASH value on day 0 (dominant/recessive model).

Statistically significant differences were demonstrated for pain experienced during elbow bending. In the dominant/recessive model, carriers of the A rs12461895 and C rs2241717 alleles experienced pain sensation more often compared to patients with other genotypes. Analogically, in the additive model, the AA, CC, and CC (rs12461895, rs4803455, rs2241717, respectively) genotypes were associated with more frequent pain sensations (Table 3). This is consistent with the previously mentioned results of PROM values and confirms the thesis that TGF-β1 affects pain sensation. The *p*-value after Hochberg correction was assessed as 0.011, designating as significant the results obtained for rs12461895 and rs2241717 variants in the dominant/recessive models.

### 2.5. TGFB1 Gene Polymorphisms and MCID

Patients who achieved minimal clinically important difference (MCID+ group) experienced significant improvement following PRP treatment, which was the opposite of patients who did not (MCID– group). Results for rs2278422 showed that CC homozygotes achieved minimal clinically important difference (MCID) more often in dominant/recessive and additive models than G allele carriers. Interestingly, the results for QDASH at week 104 showed that MCID was most likely achieved by CG heterozygotes in the additive model. Carriers of the AA genotype of rs12461895 SNP achieved MCID more often compared to the carriers of other genotypes in both models. The only exception was the results for PRTEE in week 2, in the additive model, where CC homozygotes happened more often in the MCID+ group (both in dominant/recessive and additive models). In the case of rs4803455, carriers of CC genotype achieved MCID more frequently, again in both models. Lastly, CC homozygotes of rs2241717 SNP were more likely to achieve MCID in both models, with the exception of results for PRTEE at week 2, which showed that AA homozygotes achieved MCID more often (in dominant/recessive and additive models). The statistical significance threshold was set at 0.009 after correction for multiple testing. After correction, the results for rs12461895, rs4803455, and rs2241717 (VAS at week 2) remained statistically significant. Detailed results are shown in Table 4 and Table 5.

## 3. Discussion

In the present study, it has been demonstrated that analyzed genetic variants of the *TGFB1* gene may have an impact on PRP therapy. Better effectiveness of the PRP therapy was observed in rs2278422 CC homozygotes, and this SNP showed no change in pain levels prior to treatment. The contribution of rs12461895, rs4803455, and rs2241717 polymorphisms to the therapy effectiveness is uncertain due to higher values of VAS at day 0 for carriers of the AA, CC, and CC genotypes (rs12461895, rs4803455, rs2241717, respectively). The influence of the analyzed polymorphisms on pain parameters before PRP injection may distort the obtained results. It is worth noting that this most likely results from the involvement of TGF-β1 in pain sensation. The presence of studied SNPs correlated with the results of blood morphological parameters and pain perception. Numerous significant results after applying the Hochberg correction, indicating a clear relationship between the tested SNPs (especially rs2278422) and the effectiveness of PRP treatment, support an association between the analyzed polymorphisms and PRP therapy outcomes.

**Figure 4:**
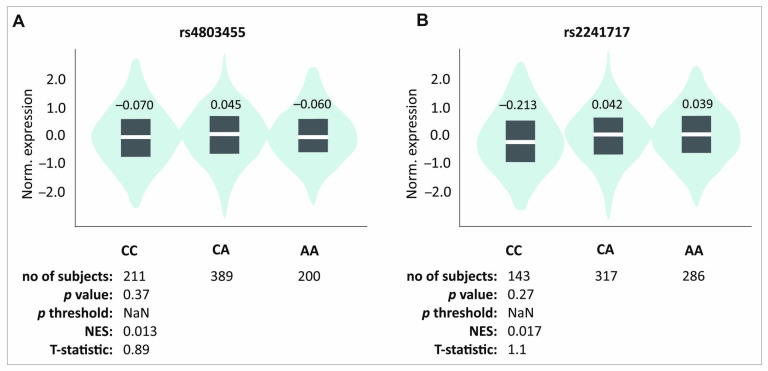
In silico analyses of the effect of (**A**) rs4803455 and (**B**) rs2241717 polymorphisms on the *TGFB1* gene expression level in whole blood. Figure prepared using GTEx portal data [31].

Rs12461895, rs4803455, and rs2241717 polymorphisms formed a haplotype; hence, the results obtained were similar. Since ΔVAS is the difference between the pain level on day 0 and a specific follow-up point, a higher VAS on day 0 experienced by the AA, CC, and CC homozygotes (rs12461895, rs4803455, and rs2241717, respectively) will modify the ΔVAS value. Interestingly, patients with these genotypes also showed higher ΔVAS values. This means that despite the stronger pain they felt before starting the therapy, they still showed improvement. Whether this improvement is actually greater than in carriers of the other genotypes remains debatable. The influence of these polymorphisms on pain sensation is also visible in other analyses. Patients with these genotypes were characterized by more frequent pain during elbow bending. These results do not seem accidental due to many reports suggesting TGF-β1 role in pain perception. As mentioned, the influence of TGF-β1 on pain may differ, probably dependently on TGF-β1-mediated signaling [21]; however, studies on human osteoporosis have shown that higher levels of TGF-β1 may alleviate pain [8]. Of the polymorphisms we analyzed, rs4803455 is the most widely studied. It has been shown that carriers of the A allele had a reduced risk of asthma [26], carotid plaque formation [27], breast and endometrial cancer [28,29], as well as post-transplant renal dysfunction [30]. However, there are no reports available in the literature presenting the rs4803455 effect on *TGFB1* expression. In silico analyses showed that CC genotypes of the rs4803455 and rs2241717 polymorphisms are associated with decreased *TGFB1* expression levels in whole blood (Figure 4). Although these results are not statistically significant, a certain trend can be observed. It appears that genotypes associated with decreased *TGFB1* expression are also associated with elevated levels of pain, which is consistent with the study of Liu et al. [8] and confirms the analgesic effect of TGF-β1 in human musculoskeletal tissues. Because PRP contains a high concentration of TGF-β1 [9], it is possible that PRP administration alleviates the pain experienced by patients. This may explain the results obtained in this study.

**Figure 5:**
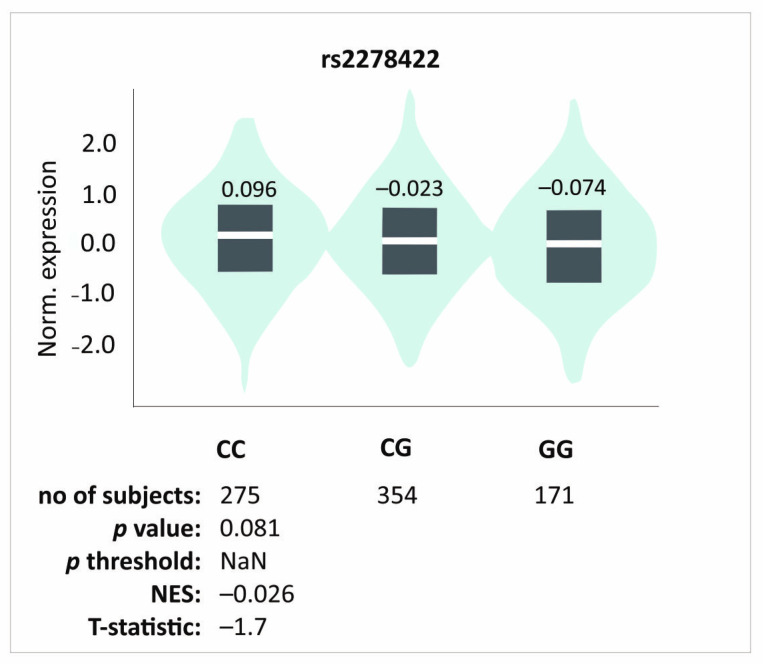
In silico analyses of the effect of rs2278422 polymorphisms on the *TGFB1* gene expression level in whole blood. Figure prepared based on GTEx portal data [31].

As previously discussed, we obtained slightly different results for rs2278422 polymorphism compared to other analyzed SNPs. This SNP is located out of the haplotype and did not show any association with pain sensation. We did not observe any differences in either PROM values before PRP injection or pain sensation during elbow bending; however, it showed an association with PRP effectiveness. Patients with the CC genotype showed better therapeutical effectiveness compared to carriers of other genotypes. Previous studies have shown that the CG genotype of this polymorphism is associated with a reduced risk of osteoarthritis [32], while GG homozygotes had a lower risk of insulin resistance [33]. No information is available in the literature about the rs2278422 effect on the expression of *TGFB1*. In silico analyses demonstrated a trend indicating increased *TGFB1* expression in the CC genotype, but the results are not statistically significant (Figure 5). Interestingly, the genotype that was associated with a better effect of PRP therapy was associated with raised levels of *TGFB1* expression. Perhaps a reduced expression level of *TGFB1* leads to increased pain, whereas the elevated expression leads to better therapeutic progress due to the involvement of TGF-β1 in the regeneration of the damaged tendon [17].

Tendinopathy is a series of changes occurring in tendons, which lead to pain and impaired function. In a damaged tendon, collagen fibers are disorganized, and ECM remodeling, proliferation, as well as angiogenesis are dysregulated [34]. It should be emphasized that many proteins can potentially affect the pathogenesis of tendinopathy. MMPs and their inhibitors have an important role in tissue remodeling [35], and polymorphisms of the *MMP3* and *TIMP2* genes are associated with the risk of Achilles tendinopathy (AT) [36]. Moreover, tenocytes and immune cells release pro-inflammatory factors in response to tissue injury, such as tumor necrosis factor (TNF), interleukin 1 beta (IL-1β), interleukin 6 (IL-6), TGF-βs, and platelet-derived growth factors (PDGFs) [34]. It was shown that polymorphisms of *IL-1β* and *IL-6* genes are also associated with AT [37]. In the same study, *COL5A1* gene SNP association with the risk of Achilles tendinopathy was also shown [37]. Since tendons are composed of collagen, it seems probable that polymorphisms in the genes encoding these proteins will also influence the pathogenesis of tendinopathy. Interestingly enough, other studies indicate that collagen has a role in pain perception as well [38]. It is important to note that factors influencing the pathogenesis of tendinopathy may also modify the effectiveness of its treatment and the level of pain. The studies we have conducted so far indicate that polymorphisms of genes encoding cytokines from the PDGF/VEGF superfamily [39,40,41,42] and PDGF receptors (PDGFR) [43,44] are associated with the effectiveness of PRP, while *COL1A1* gene SNPs showed association mainly with pain sensations [45]. This indicates a wide range of factors potentially modulating the effectiveness of tendinopathy treatment using PRP therapy, which results from the fact that many factors are involved in its pathogenesis. Moreover, the growth factors and their receptors that we have studied previously can interact with each other and with TGF-β1. It has been shown that VEGFA can bind to PDGFR [46], while PDGFRB interacts with TGF-beta signaling [47]. This adds an additional layer of complexity to the factors potentially modulating the treatment of tendinopathy and their interactions.

The current work also provides interesting observations of the analyzed TGF-β1 variant’s association with the results of blood morphological parameters. Investigated polymorphisms showed an association, among others, with MPV, PLT, PDW, NEU, EOS, and MONO levels. Those associations may be justified by the participation of TGF-β1 in hematopoiesis. In vitro studies have shown that TGF-β1 inhibits hematopoiesis [48,49,50]; however, the functions of TGF-β1 are largely determined by the environment, cell type, and differentiation level [51,52]. In vivo studies of TGF-β1 in a mouse model have shown that loss of this protein leads to a decrease in proteins indispensable for hemopoiesis, which may cause its defects [49]. Furthermore, TGF-β1 determines hematopoietic stem cell (HSC) fate and maintains the self-renewal ability of HSCs in vivo [48]. This indicates a significant and not fully understood role of TGF-β1 in the regulation of hematopoiesis.

Rs2278422, rs4803455, and rs2241717 showed an association with platelet parameters, which is particularly important in the context of PRP effectiveness. Although these results were not statistically significant after correction, a certain trend was observed. In the dominant/recessive model, the C allele of the rs2278422 polymorphism, which was associated with a better effect of PRP therapy, was also associated with increased values of MPV. This is consistent with our previous results indicating that larger platelets are more active, which positively translates into PRP activity [43]. Moreover, the AA genotype of rs2241717 was associated with higher PLT levels and the C allele of rs4803455 with higher PDW (Table 2). Interestingly, studies indicate that TGF-β1 inhibits megakaryopoiesis [53,54,55,56]. Since TGF-β1 is produced in large amounts by megakaryocytes and platelets, this growth factor could be considered a possible feedback regulator of megakaryopoiesis [53,56]. However, studies in TGF-β1 knockout mice have shown that loss of TGF-β1 function leads to a decrease in the amount of megakaryocyte–erythroid progenitor cells (MEPs) [49]. There is a lack of information in the literature on the effect of TGF-β1 on platelet parameters; therefore, we do not know how polymorphisms investigated in this study could affect the MPV, PLT, and PDW parameters. Moreover, due to the bifunctional action of TGF-β1 and the above-mentioned studies on TGF-β1-deficient mice, it is difficult to definitively determine how TGF-β1 affects platelet formation.

Moreover, analyzed polymorphisms also showed an association with the results of monocytes, eosinophils, and neutrophils levels. The literature data indicate that TGF-β1 may influence neutrophil signaling and gene expression but not migration [57], inhibit eosinophils recruitment [58] and induce their degranulation [59], as well as promote the maturation and differentiation of monocytic cells [52]. It was shown that loss of TGF-β1 function also led to a decrease in the number of granulocyte–monocyte progenitor cells (GMPs) [49], which can affect neutrophil, eosinophil, and monocyte levels. In our study, the AC heterozygotes (additive model) and carriers of the C allele (dominant/recessive model) of rs4803455 had higher levels of NEU, and the AC genotype was associated with the highest level of *TGFB1* expression in in silico analysis. Carriers of these genotypes also had lower EOS percentages (additive model), which may be associated with TGF-β1’s role in eosinophil recruitment inhibition. In the case of monocytes, the C allele of rs224171, which was associated with lower *TGFB1* expression, was also associated with lower levels of MONO (Table 2, Supplementary Table S1, Figure 4). Circumstantial information available in the literature is consistent with our results; however, determining the influence of the studied SNPs on blood morphological parameters requires further studies. Due to the questionable influence of rs4803455 and rs224171 polymorphisms on the effectiveness of PRP therapy, it is also difficult to determine whether these changes in the patients’ blood counts could affect the effectiveness of the treatment.

The main limitations of our study are the relatively small study group and the lack of restrictions on other forms of therapy after PRP injection. The lack of limitations for other forms of therapy results from ethical issues. In our opinion, denying access to other forms of treatment for two years to patients for whom PRP therapy was not effective is unethical. Moreover, to reduce the risk of falsely positive results, we performed both quantitative and qualitative analysis and applied a correction for multiple tests. Nevertheless, confirmation of the obtained results requires more detailed research on a larger study group. In particular, determining the influence of the analyzed SNPs on the *TGFB1* expression would enable a better understanding of their exact function.

## 4. Materials and Methods

### 4.1. Study Design

This study was performed in accordance with STROBE and MIBO guidelines. The 1975 Declaration of Helsinki and its subsequent amendments were followed. Written informed consent was obtained from each participant, and the study protocol received approval from the Ethics Committee of the Silesian Medical University in Katowice (KNW/0022/KB1/24/I/17). This research used the same effectiveness measures, follow-up timeline, patient selection rules, PRP separation, and injection procedure, as well as blood analysis as in our previous studies [39,43]. Blood morphology and genetic analyses of selected SNPs of the *TGFB1* gene were performed.

### 4.2. Measures of Effectiveness and Follow-Up

Patients’ therapeutic progress was documented over a two-year period at 2, 4, 8, 12, 24, 52, and 104 weeks after PRP injection. The effectiveness was measured using minimal clinically important difference and patient-reported outcome measures, specifically VAS, QDASH, and PRTEE. QDASH and PRTEE questionnaires were translated and culturally adapted for Polish patients [60,61]. The rating scale was 0 for minimum and 10 for maximum pain for VAS, 0 for minimum, and 100 for maximum pain and disability for QDASH and PRTEE. Therapeutic progress scores were compared for each patient to the baseline on the day of PRP injection (baseline at week 0) to calculate ΔVAS, ΔQDASH, and ΔPRTEE values. Based on the current literature, point values were determined to indicate achievement of MCID: 1.5 points for VAS [62], 15.8 points for QDASH [63], and 11 points for PRTEE [64]. This score was used to assess MCID for all PROMs at each follow-up point. Patients were assigned to the MCID+ group when they achieved MCID or MCID− when they did not. In order to verify whether confounding factors such as comorbidities or lifestyle affect the achievement of MCID, we conducted additional statistical analyses. In the study group, only vitamin B12 supplementation (QDASH week 52) and gout (PRTEE week 12) were found to be associated with the achievement of MCID. Other comorbidities or factors, such as smoking or alcohol consumption, did not affect the achievement of MCID.

### 4.3. Patient Selection and Characteristics of the Study Group

**Figure 6:**
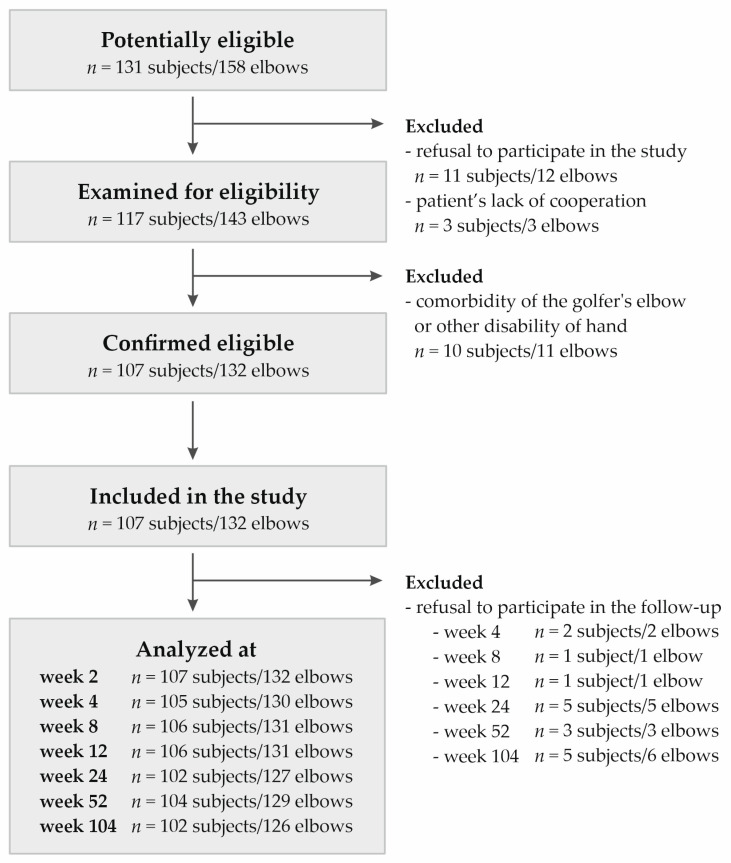
Flow chart presenting selection of studied group.

Recruitment of the group of patients eligible for this study was conducted from November 2018 to November 2019, including data collection until November 2021. This study included 107 Polish Caucasian participants from Upper Silesia (132 elbows with 25 bilateral cases). The group consisted of 65 women and 42 men between the ages of 24 and 64 years (median ± QD: 46.00 ± 5.50). All patients were diagnosed with lateral elbow tendinopathy, classified under the ICD-10 code M77.1. The inclusion criteria were tenderness on palpation of the lateral epicondyle, weakness of grip strength, morning stiffness, as well as positive Thomson, Mill, and Cozen tests. Exclusion criteria were the presence of other diseases or injuries (such as rheumatoid arthritis, active cancer, cervical radiculopathy), previous PRP injections, pregnancy, prior surgical treatments, and use of antiplatelet drugs or steroid injections in the last 6 months. Complementary therapies, such as NSAIDs, steroids, physiotherapy, and subsequent PRP injections, were monitored but not considered part of the exclusion criteria. Figure 6 shows the selection of the study group.

Before the PRP injection (week 0), pain characteristics were examined, such as pain radiating from the lateral epicondyle of the humerus to the wrist, shoulder, or neck, as well as pain increasing when holding, lifting, or grabbing objects. Comorbidities were observed in the study group, and the most common were thyroid disease, hypertension, and gout. The mean WBC concentration was 6.26 ± 1.16 (10^9^/L ± QD); PLT count was 240.00 ± 40.50 (10^9^/L ± QD), and MPV was 9.10 ± 0.73 (fL ± QD). In whole blood, females had higher levels of platelet (261.50 ± 33.00 vs. 224.00 ± 38.75, respectively, *p* = 0.000) and plateletcrit (2.37 ± 0.36 vs. 2.04 ± 0.33, respectively, *p* = 0.001) compared to males. Patients experienced pain most frequently when lifting, which radiated mainly to the forearm (Table 6).

### 4.4. PRP Separation, Injection Procedure, Whole Blood, and PRP Parameters

Twelve mL of whole blood was obtained from each patient under standardized conditions in the treatment room, using disposable materials. An Arthrex Autologous Conditioned Plasma double syringe from Arthrex GmbH, Munich, Germany, was used to extract the plasma. Immediately after blood collection, PRP was separated. The collected blood was mixed with 3.13% sodium citrate (MediPac^®^ GmbH, Königswinter, Germany) at a ratio of 9:1 and then centrifuged in a Rotofix 32A centrifuge (Andreas Hettich GmbH & Co., Tuttlingen, Germany) at 1500 rpm for 5 min. For patients with bilateral elbow tendinopathy, PRP was obtained from each elbow. Immediately after PRP was obtained, it was injected in a volume of 2.0–3.0 mL using a 1.2 mm needle into the common extensor attachment region under ultrasound guidance (Mindray DC-3, Mindray Medical Poland Sp. z o.o., Warsaw, Poland). To monitor potential complications after the PRP injection procedure, patients were kept under observation for 30 min. With no worrisome symptoms noted, patients were discharged and informed to contact the hospital if side effects occurred.

A complete blood count was performed on the same day. Laboratory parameters included WBC, RBC, hemoglobin (HGB), hematocrit (HCT), mean corpuscular volume (MCV), mean corpuscular hemoglobin concentration (MCH), MCHC, red blood cell distribution width (RDW), PLT, MPV, PDW, PCT, NEU, LYM, MONO, EOS, and basophil (BASO). Platelet-related parameters such as platelet count, PCT, MPV, and PDW in fresh PRP were also evaluated.

### 4.5. Genetic Analyses

**Figure 7:**
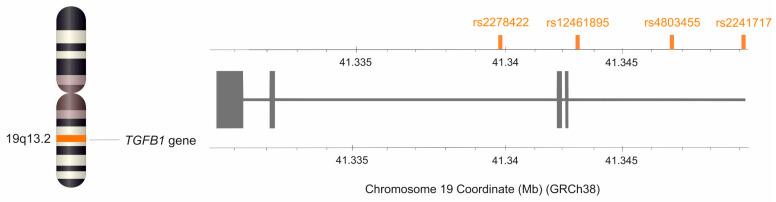
Location of the *TGFB1* gene on chromosome 19 and location of the studied SNPs. The figure was created using the data from LD-matrix tool [65].

For all the SNPs of the *TGFB1* gene selected for analysis, the minor allele frequency in the European population was ≥20%, according to information in the SNP database of the National Center for Biotechnology Information, US National Library of Medicine [25]. This study included rs2278422 (C>G), rs12461895 (C>A), rs4803455 (C>A), and rs2241717 (A>C) variants (Figure 7). Isolation of genomic DNA was performed from peripheral blood leukocytes using the MasterPure DNA purification kit (Epicenter Technologies, Madison, WI, USA). Genotyping of selected polymorphisms of the *TGFB1* gene was performed using TaqMan Predesigned SNP Genotyping Assay kits (Thermo Fisher Scientific, Waltham, MA, USA) and the LightCycler^®^480 Real-Time PCR System (F. Hoffmann-La Roche AG, Basel, Switzerland). To confirm the accuracy of genotyping, the test was repeated for 15% of samples, and the repeatability was 100%. Genotyping was unsuccessful for five patients during analyses of rs2241717 polymorphism.

### 4.6. Statistical Analysis

Statistical analysis of the obtained results was performed using Statistica 13.0 (TIBCO Software Inc., Palo Alto, CA, USA). The normality distribution was assessed using the Shapiro–Wilk test. All quantitative variables showed a non-normal distribution, and therefore, the Mann–Whitney U test and the Kruskal–Wallis test were used for comparisons. Quantitative results were reported as median with interquartile range (QD). Statistically significant results were considered those with *p* < 0.05. For multiple comparisons, *p*-values were adjusted using the Hochberg correction [66]. Genetic data were analyzed using dominant/recessive and additive models of inheritance. Using the χ^2^ test, the Hardy–Weinberg equilibrium was assessed, and qualitative data were compared. Yates’ correction was used for subgroups with less than 10 patients. HaploView 4.2 software (Broad Institute of MIT and Harvard, Cambridge, MA, USA) [67] was used to identify haplotype blocks, according to the algorithm of Gabriel et al. [68]. Linkage disequilibrium was assessed using D′ and R^2^ values.

## 5. Conclusions

In conclusion, our results indicate that rs2278422 may affect the effectiveness of PRP therapy, whereas rs12461895, rs4803455, and rs2241717 are associated primarily with pain experienced by patients before therapy. The PRP therapy was more effective for patients with the CC rs2278422 genotype, while AA, CC, and CC homozygotes (rs12461895, rs4803455, and rs2241717, respectively) experienced more severe pain before therapy. TGF-β1 is involved in regeneration, wound and fracture healing, as well as tendon development and maturation, which is probably the reason for its positive effect on PRP effectiveness. In turn, reduced expression of *TGFB1* may be associated with increased pain due to the analgesic effect of this protein. The exact effect of TGF-β1 on pain sensation is not determined, but studies on human osteoporosis are consistent with the results of the present study. Moreover, all analyzed polymorphisms showed associations with different blood morphological parameters, which is probably due to the participation of TGF-β1 in hematopoiesis. However, due to the bifunctional effect of this protein, it is difficult to clearly determine the impact of the analyzed SNPs on patients’ blood morphological parameters.

## Figures and Tables

**Table 1:** The studied polymorphisms of the *TGFB1* gene, their chromosomal location, and frequency, along with results for the Hardy–Weinberg test.

*TGFB1* SNP	Chromosomal Localization *	Alleles	n	%	Genotypes	n	%	Minor Allele	Population MAF **	HWE *p*-Value
rs2278422	19:41339853	C	103	54.21	CC	45	34.09	G	42.9	0.462
		G	87	45.79	GC	58	43.94			
					GG	29	21.97			
rs12461895	19:41342442	C	101	49.75	CC	30	22.73	A	41.4	0.684
		A	102	50.25	AC	71	53.79			
					AA	31	23.48			
rs4803455	19:41345604	C	116	54.72	CC	36	27.27	A	47.5	0.022
		A	96	45.28	AC	80	60.61			
					AA	16	12.12			
rs2241717	19:41348147	A	95	48.97	AA	28	22.05	C	43.9	0.818
		C	99	51.03	AC	67	52.76			
					CC	32	25.20			

Legend: SNP, single-nucleotide polymorphism; MAF, minor allele frequency; HWE, Hardy–Weinberg equilibrium. * GRCh38, chromosome 19, ** 1000 genomes, Europe.

**Table 2:** Association of analyzed *TGFB1* gene polymorphisms and blood morphological parameters in a dominant/recessive model.

SNP	Blood Morphological Parameters	Genotypes				*p*-Value
		Median	QD	Median	QD	
rs2278422		GG		CG/CC		
	NEU [%]	57.60	4.55	63.40	5.45	0.011
	NEU [10^9^/L]	3.29	1.07	4.14	1.04	0.041
	LYM [%]	34.00	6.95	28.00	4.30	0.009
		CC		CG/GG		
	MPV [fL]	9.65	0.75	9.00	0.70	0.021
	NEU [10^9^/L]	4.36	0.80	3.39	0.88	0.026
	MPV PRP [fL]	8.80	0.45	8.50	0.45	0.018
rs12461895		CC		AC/AA		
	MCHC [g/dL]	32.60	0.47	32.90	0.50	0.010
	PCT [mL/L]	2.49	0.27	2.16	0.31	0.026
	MONO [%]	5.95	2.10	4.60	1.20	0.017
	MONO [10^9^/L]	0.36	0.10	0.30	0.06	0.018
		AA		AC/CC		
	EOS [%]	2.75	1.13	2.10	1.00	0.028
rs4803455		CC		AC/AA		
	PDW [fL]	16.20	0.15	16.00	0.20	0.027
		AA		AC/CC		
	WBC [10^9^/L]	5.28	0.92	6.36	1.16	0.049
	RBC [10^12^/L]	4.87	0.32	4.66	0.28	0.024
	NEU [%]	56.50	3.35	62.90	5.45	0.002 *
	NEU [10^9^/L]	2.76	0.12	4.14	0.92	0.004 *
	EOS [%]	2.60	1.25	2.10	1.00	0.039
	LYM [%]	32.60	3.80	28.00	5.40	0.018
	MONO [%]	6.40	1.65	4.70	1.30	0.027
rs2241717		CC		AC/AA		
	PDW [fL]	16.20	0.10	16.00	0.20	0.029
	EOS [%]	2.70	1.10	2.05	1.00	0.021
	EOS [10^9^/L]	0.17	0.04	0.12	0.06	0.050
		AA		AC/CC		
	MCHC [g/dL]	32.60	0.50	32.90	0.50	0.011
	PLT [10^9^/L]	259.50	33.00	229.00	37.50	0.040
	PCT [mL/L]	2.53	0.18	2.20	0.34	0.010
	MONO [%]	6.20	1.87	4.70	1.25	0.003 *
	MONO [10^9^/L]	0.38	0.09	0.30	0.08	0.002 *

Legend: SNP, single-nucleotide polymorphism; QD, quartile deviation; NEU, neutrophils; LYM, lymphocytes; MPV, mean platelet volume; PRP, platelet-rich plasma; MCHC, mean corpuscular hemoglobin concentration; PCT, plateletcrit; MONO, monocytes; EOS, eosinophils; PDW, platelet distribution width; WBC, white blood cells; RBC, red blood cells. * statistically significant after Hochberg correction (threshold of significance: *p* ≤ 0.004).

**Table 3:** The distribution of genotype frequencies of the *TGFB1* gene polymorphisms for patients experiencing pain during elbow bending (in additive and dominant/recessive models).

Model of Inheritance	SNP	Genotype	Presence of Pain		No Pain		*p*-Value
			n	%	n	%	
Additive	rs12461895	CC	15	16.30	14	36.84	0.031
		AC	52	56.52	18	47.37	
		AA	25	27.17	6	15.79	
	rs4803455	CC	30	32.61	6	15.79	0.030
		AC	55	59.78	24	63.16	
		AA	7	7.61	8	21.05	
	rs2241717	AA	13	14.17	14	37.84	0.013
		AC	49	55.68	17	45.95	
		CC	26	29.55	6	16.22	
Dominant/recessive	rs12461895	CC	15	16.30	14	36.84	0.011 *
		AA/AC	77	83.70	24	63.13	
	rs2241717	AA	13	14.77	14	37.84	0.004 *
		AC/CC	75	85.23	23	62.16	

Legend: SNP, single-nucleotide polymorphism. * statistically significant differences after Hochberg correction (threshold of significance: *p* ≤ 0.011).

**Table 4:** The distribution of genotype frequencies of the *TGFB1* gene polymorphisms in additive model for patients who did (MCID+) and did not (MCID−) achieve MCID threshold in PROM values after PRP therapy.

SNP	PROM	Week	Genotype												*p*-Value
			MCID+ Patients						MCID− Patients						
			n	%	n	%	n	%	n	%	n	%	n	%	
rs2278422			CC		CG		GG		CC		CG		GG		
	VAS	24	36	83.72	31	56.36	19	65.52	7	16.28	24	43.64	10	34.48	0.015
	QDASH	104	26	63.41	46	83.64	15	55.56	15	36.59	9	16.36	12	44.44	0.014
	PRTEE	2	34	79.07	36	62.07	15	51.72	9	20.93	22	37.93	14	48.28	0.044
rs12461895			CC		AC		AA		CC		AC		AA		
	VAS	2	11	37.93	28	40.00	22	70.97	18	62.07	42	60.00	9	29.03	0.009 *
		4	18	62.07	36	51.43	25	80.65	11	37.93	34	48.57	6	19.35	0.021
		24	20	68.97	39	58.21	27	87.10	9	31.30	28	41.79	4	12.90	0.017
	PRTEE	2	25	86.21	40	57.41	20	64.52	4	13.79	30	42.68	11	35.48	0.014
rs4803455			CC		AC		AA		CC		AC		AA		
	VAS	2	24	66.67	30	38.46	7	43.75	12	33.33	48	61.54	9	56.25	0.019
	QDASH	52	26	74.29	49	62.82	6	37.50	9	25.71	29	37.18	10	62.50	0.042
rs2241717			AA		AC		CC		AA		AC		CC		
	VAS	2	11	40.74	26	39.39	22	68.75	16	59.26	40	60.61	10	31.25	0.018
		4	16	59.26	34	51.52	25	78.13	11	40.74	32	48.48	7	21.88	0.041
		24	18	66.67	38	57.14	27	84.83	9	33.33	27	42.86	5	15.63	0.029
	PRTEE	2	23	85.19	38	57.78	20	62.50	4	14.81	28	42.42	12	37.50	0.039

Legend: SNP, single-nucleotide polymorphism; PROM, patient-reported outcome measures; MCID, minimal clinically important difference. * statistically significant differences after Hochberg correction (threshold of significance: *p* ≤ 0.009).

**Table 5:** Genotype frequencies of the *TGFB1* gene polymorphisms for patients who did (MCID+) and did not (MCID−) achieve MCID threshold in PROM values after PRP therapy (dominant/recessive model).

SNP	PROM	Week	Genotype								*p*-Value
			MCID+ Patients				MCID− Patients				
			n	%	n	%	n	%	n	%	
rs2278422			CC		CG/GG		CC		CG/GG		
	VAS	8	33	75.00	47	54.02	11	25.00	40	45.98	0.020
		24	36	83.72	50	59.52	7	16.28	34	40.48	0.010
	PRTEE	2	34	79.07	51	58.62	9	20.93	36	41.38	0.035
rs12461895			AA		AC/CC		AA		AC/CC		
	VAS	2	22	70.97	39	39.39	9	29.03	60	60.61	0.004 *
		4	25	80.65	54	54.55	6	19.35	45	45.45	0.017
		24	27	87.10	59	61.46	4	12.90	37	38.54	0.015
			CC		AC/AA		CC		AC/AA		
	PRTEE	2	25	86.12	60	59.14	4	13.79	41	40.59	0.014
rs4803455			CC		AC/AA		CC		AC/AA		
	VAS	2	24	66.67	37	39.36	12	33.33	57	60.64	0.005 *
		24	30	83.33	56	61.54	6	16.67	35	38.46	0.031
rs2241717			CC		AC/AA		CC		AC/AA		
	VAS	2	22	68.75	37	39.78	10	31.25	56	60.22	0.009 *
		4	25	78.13	50	53.76	7	21.88	43	46.24	0.027
		24	27	84.38	54	60.00	5	15.63	36	40.00	0.022
			AA		AC/CC		AA		AC/CC		
	PRTEE	2	23	85.19	58	59.18	4	14.81	40	40.82	0.023

Legend: SNP, single-nucleotide polymorphism; PROM, patient-reported outcome measures; MCID, minimal clinically important difference. * statistically significant differences after Hochberg correction (threshold of significance: *p* ≤ 0.009).

**Table 6:** General characteristics of the study group on baseline (week 0).

Characteristics			
General	number of subjects, N	107	-
	number of elbows, n (%)	132	(100.00)
	age, median ± QD	46.00	5.50
	BMI, median ± QD	25.65	2.00
	current smokers, n (%)	22	(16.67)
Comorbidities	diabetes mellitus, n (%)	4	(3.03)
	gout, n (%)	8	(6.06)
	obesity (BMI ≥ 30), n (%)	26	(19.70)
	overweight/obesity (BMI ≥ 25), n (%)	86	(65.15)
	hypercholesterolemia, n (%)	10	(7.58)
	hypertension, n (%)	18	(13.64)
Pain of elbow	in LE area, n (%)	132	(100.00)
	during the day, n (%) *	92	(70.80)
	at night, n (%) *	67	(51.54)
	during lifting, n (%) *	117	(90.00)
	when grabbing, n (%) *	80	(61.54)
	when pressing, n (%) *	85	(65.39)
	when bending the elbow, n (%) *	92	(70.77)
	when bending the wrist, n (%) *	44	(33.85)
Pain radiating to the	wrist, n (%) *	40	(30.77)
	forearm, n (%) *	65	(50.00)
	arm, n (%) *	32	(24.62)
	shoulder, n (%) *	26	(20.00)
	neck, n (%) *	12	(9.23)

Legend: BMI, body mass index; LE, lateral epicondyle of the humerus; QD, quartile deviation; * data available for 130 elbows.

## Data Availability

The original contributions presented in this study are included in this article/Supplementary Materials; further inquiries can be directed to the corresponding author.

## Supplementary Materials

The following supporting information can be downloaded at https://www.mdpi.com/article/10.3390/ijms26062431/s1.
