# Supplementary material for: Association of the TGFB1 Gene Polymorphisms with Pain Symptoms and the Effectiveness of Platelet-Rich Plasma in the Treatment of Lateral Elbow Tendinopathy: A Prospective Cohort Study
Source: Int J Mol Sci. 2025 Mar 8;26(6):2431. doi: 10.3390/ijms26062431 (PMC11942043; doi:10.3390/ijms26062431)
Supplement: Supplementary file 1 [file ijms-26-02431-s001.zip › Supplementary Table S1.pdf]

Table S1. Median (±QD) of blood morphological parameters for genotypes of the *TGFB1* gene polymorphisms in the additive model.

Blood morphological parameters	Genotype of rs2278422						*p* value			
	CC		CG		GG					
	Median	±QD	Median	±QD	Median	±QD	Kruskal-Wallis test	CC vs CG	CC vs GG	CG vs GG
MPV [fl]	9.65	0.75	8.90	0.45	9.40	1.00	0.017	**0.016**	1.000	0.273
NEU [%]	64.15	4.25	62.30	5.65	57.60	4.55	0.034	1.000	**0.034**	0.123
NEU [10*9/L]	4.36	0.80	3.75	0.82	3.29	1.07	0.039	0.379	**0.036**	0.637
LYM [%]	27.50	4.80	28.20	5.20	34.00	6.95	0.032	1.000	**0.037**	0.086

Blood morphological parameters	Genotype of rs12461895						*p* value			
	AA		AC		CC					
	Median	±QD	Median	±QD	Median	±QD	Kruskal-Wallis test	AA vs AC	AA vs CC	AC vs CC
MCHC [g/dl]	33.10	0.52	32.85	0.50	32.60	0.47	0.037	1.000	0.204	**0.036**
EOS [%]	2.75	1.13	1.80	0.95	2.30	1.10	0.039	**0.040**	0.898	0.576

Blood morphological parameters	Genotype of rs4803455						*p* value			
	AA		AC		CC					
	Median	±QD	Median	±QD	Median	±QD	Kruskal-Wallis test	AA vs AC	AA vs CC	AC vs CC
NEU [%]	56.50	3.35	63.20	4.50	62.10	5.95	0.003*	**0.003**	0.125	0.508
NEU [10*9/L]	2.76	0.12	4.28	0.93	3.57	0.72	0.008*	**0.007**	0.199	0.551
EOS [%]	2.60	1.25	1.80	0.85	2.70	1.25	0.009*	**0.037**	1.000	0.065
LYM [%]	32.60	3.80	27.75	5.03	28.40	5.10	0.026	**0.030**	0.488	0.531

Blood morphological parameters	Genotype of rs2241717						*p* value			
	AA		AC		CC					
	**Median**	**±QD**	**Median**	**±QD**	**Median**	**±QD**	**Kruskal-Wallis test**	**AA vs AC**	**AA vs CC**	**AC vs CC**
MCHC [g/dl]	32.60	0.50	32.90	0.50	33.10	0.50	0.036	**0.033**	0.215	1.000
EOS [%]	2.20	1.30	1.80	0.70	2.70	1.10	0.022	0.397	1.000	**0.023**
EOS [10*9/L]	0.15	0.07	0.12	0.07	0.17	0.04	0.033	0.256	1.000	**0.049**
MONO [%]	6.20	1.78	4.60	1.35	4.70	1.00	0.013	**0.012**	0.079	1.000
MONO [10*9/L]	0.38	0.09	0.29	0.08	0.31	0.06	0.009*	**0.010**	**0.043**	1.000

Legend: *TGFB1*, transforming growth factor beta1; QD, quartile deviation; EOS, eosinophils; NEU, neutrophils; LYM, lymphocytes; MCHC, mean corpuscular hemoglobin concentration; MPV, mean platelet volume ; MONO, monocytes. * - statistically significant after Hochberg correction ($p \leq 0.009$).
