# Supplementary material for: Association of the TGFB1 Gene Polymorphisms with Pain Symptoms and the Effectiveness of Platelet-Rich Plasma in the Treatment of Lateral Elbow Tendinopathy: A Prospective Cohort Study
Source: Int J Mol Sci. 2025 Mar 8;26(6):2431. doi: 10.3390/ijms26062431 (PMC11942043; doi:10.3390/ijms26062431)
Supplement: Supplementary file 1 [file ijms-26-02431-s001.zip › Supplementary Table S2.pdf]

Table S2. PROMs values (median ± QD) in carriers of different genotypes of the rs2278422 polymorphism of the *TGFB1* gene (dominant/recessive model).

PROMs values in GG homozygotes and C allele carriers of the rs2278422 *TGFB1* gene polymorphism.

**PROMs**		**GG rs2278422**		**CG+CC rs2278422**		***p***
	week	median	± QD	median	± QD	**Mann-Whitney U test**
VAS	0	5.00	2.00	6.00	1.50	0.865
	2	3.00	1.00	4.00	1.50	0.210
	4	3.00	1.50	3.00	1.50	0.298
	8	4.00	2.00	3.00	1.50	0.250
	12	3.00	1.50	2.00	1.50	0.454
	24	3.00	2.00	2.00	2.00	0.655
	52	1.00	2.00	2.00	2.00	0.582
	104	1.00	1.50	1.00	1.50	0.531
ΔVAS (vs week 0)	2	2.00	2.00	1.00	1.50	0.265
	4	2.00	2.00	2.00	2.00	0.511
	8	2.00	2.50	3.00	2.00	0.261
	12	4.00	2.50	3.00	2.00	0.785
	24	3.00	2.00	3.00	2.00	0.751
	52	4.00	2.50	3.25	2.00	0.680
	104	5.00	3.00	4.00	2.00	0.690
QDASH	0	47.27	13.64	52.27	12.05	**0.025**
	2	36.36	13.64	39.77	15.91	0.311
	4	31.82	12.50	37.50	14.77	0.275
	8	34.09	18.18	31.82	18.18	0.355
	12	27.27	12.50	29.55	19.32	0.818
	24	25.00	18.18	25.00	21.59	0.977
	52	13.64	19.32	20.45	22.73	0.305
	104	13.64	21.59	13.64	20.45	0.736
ΔQDASH (vs week 0)	2	2.27	13.64	6.82	12.50	0.170
	4	4.54	14.77	15.90	13.75	0.125
	8	4.54	17.96	18.18	17.05	**0.017**
	12	18.18	17.05	19.32	17.05	0.202
	24	15.91	14.77	21.59	19.32	0.109
	52	22.72	20.45	21.59	19.38	0.457
	104	20.45	23.86	34.09	18.75	0.057
PRTEE	0	45.50	15.50	53.00	14.00	0.170
	2	27.00	9.50	31.00	17.75	0.520
	4	24.00	12.50	25.75	14.25	0.268
	8	24.00	16.75	22.00	14.75	0.491
	12	21.00	14.50	20.00	15.00	0.840
	24	15.00	11.50	14.50	18.00	0.839
	52	7.50	10.25	13.00	15.75	0.267
	104	7.00	10.50	7.50	14.00	0.426
ΔPRTEE (vs week 0)	2	11.00	12.25	17.00	10.00	0.205
	4	17.00	21.75	22.00	12.50	0.529
	8	22.50	20.75	28.25	14.25	0.099
	12	29.50	21.50	28.50	14.75	0.458
	24	28.50	15.75	31.25	19.25	0.646
	52	33.50	18.25	32.75	18.13	0.833
	104	34.00	18.50	38.75	16.13	0.541

PROMs values in CC homozygotes and G allele carriers of the rs2278422 *TGFB1* gene polymorphism.

**PROMs**		**CC rs2278422**		**CG + GG rs2278422**		***p***
	week	median	± QD	median	± QD	**Mann-Whitney U test**
VAS	0	6.00	2.00	6.00	1.50	0.619
	2	4.00	1.75	4.00	1.50	0.917
	4	3.00	1.75	3.00	1.50	0.177
	8	2.00	1.50	4.00	2.00	**0.030**
	12	1.00	1.50	3.00	2.00	**0.028**
	24	1.00	1.50	3.00	2.00	**0.007***
	52	1.00	1.50	2.00	2.50	0.087
	104	1.00	1.50	1.00	1.50	0.707
ΔVAS (vs week 0)	2	1.00	1.50	1.00	1.50	0.855
	4	2.00	1.50	2.00	2.00	0.165
	8	3.00	1.75	2.00	2.00	**0.040**
	12	3.00	2.00	2.00	2.00	**0.042**
	24	4.00	2.00	2.00	1.50	**0.004***
	52	4.00	2.50	2.00	2.00	**0.045**
	104	4.00	2.50	4.00	2.00	0.548
QDASH	0	54.54	11.37	50.00	13.64	0.636
	2	39.77	18.35	38.64	13.64	0.967
	4	32.95	16.48	36.36	13.64	0.402
	8	25.00	17.05	36.36	17.05	0.062
	12	22.73	15.91	31.82	19.32	**0.027**
	24	13.64	19.32	28.41	21.02	**0.030**
	52	15.91	22.73	20.45	22.73	0.148
	104	10.23	21.59	13.64	14.77	0.731
ΔQDASH (vs week 0)	2	6.82	17.39	6.81	13.63	0.664
	4	13.63	18.18	11.36	17.04	0.269
	8	17.04	19.89	11.36	18.18	0.078
	12	27.27	24.55	15.91	14.77	0.056
	24	38.63	24.41	18.17	16.48	**0.041**
	52	27.27	20.46	20.45	18.18	0.122
	104	29.54	23.87	30.68	20.45	0.930
PRTEE	0	53.50	13.00	51.50	14.50	0.767
	2	26.00	16.63	30.50	16.75	0.441
	4	20.25	15.63	26.50	13.50	0.342
	8	15.50	10.75	27.50	17.50	**0.027**
	12	13.50	12.25	23.00	17.75	**0.050**
	24	9.50	12.13	19.50	17.13	**0.030**
	52	11.00	10.25	13.00	17.50	0.182
	104	7.25	14.75	7.25	10.50	0.787
ΔPRTEE (vs week 0)	2	18.00	8.00	13.50	14.75	0.201
	4	21.00	15.50	21.50	13.25	0.672
	8	31.25	14.63	24.50	16.25	0.069
	12	29.75	13.75	26.50	16.95	0.132
	24	35.50	18.75	27.20	16.38	0.064
	52	36.25	15.25	32.00	17.75	0.176
	104	37.00	15.75	38.50	16.50	0.506

Legend: *TGFB1*, transforming growth factor beta 1; QD, quartile deviation; VAS, visual analog scale; QDASH, quick version of disabilities of the arm, shoulder and hand score; PRTEE, patient-rated tennis elbow evaluation; PROM, patient-reported outcome measures. *statistically significant after Hochberg correction ($p \leq 0.007$).
