# Supplementary material for: Association of the TGFB1 Gene Polymorphisms with Pain Symptoms and the Effectiveness of Platelet-Rich Plasma in the Treatment of Lateral Elbow Tendinopathy: A Prospective Cohort Study
Source: Int J Mol Sci. 2025 Mar 8;26(6):2431. doi: 10.3390/ijms26062431 (PMC11942043; doi:10.3390/ijms26062431)
Supplement: Supplementary file 1 [file ijms-26-02431-s001.zip › Supplementary Table S3.pdf]

**Table S3.** PROMs values (median ± QD) in carriers of different genotypes of the rs12461895 polymorphism of the *TGFB1* gene (dominant/recessive model).

PROMs values in CC homozygotes and A allele carriers of the rs12461895 *TGFB1* gene polymorphism.

| **PROMs** | | **CC rs12461895** | | **AC+AA rs12461895** | | ***p*** |
|---|---|---|---|---|---|---|
| | week | median | ± QD | median | ± QD | **Mann-Whitney U test** |
| VAS | 0 | 6.00 | 1.50 | 6.00 | 2.00 | 0.723 |
| | 2 | 4.00 | 1.50 | 4.00 | 1.50 | 0.598 |
| | 4 | 3.00 | 2.00 | 3.00 | 1.50 | 0.367 |
| | 8 | 3.00 | 2.00 | 3.00 | 2.00 | 0.576 |
| | 12 | 2.50 | 1.50 | 2.50 | 1.50 | 0.789 |
| | 24 | 3.00 | 2.00 | 2.00 | 2.00 | 0.905 |
| | 52 | 2.00 | 1.50 | 1.00 | 2.00 | 0.459 |
| | 104 | 1.00 | 1.50 | 1.00 | 1.50 | 0.856 |
| ΔVAS (vs week 0) | 2 | 1.00 | 1.50 | 1.00 | 1.50 | 0.772 |
| | 4 | 2.00 | 1.50 | 2.00 | 2.00 | 0.841 |
| | 8 | 2.00 | 1.25 | 3.00 | 2.50 | 0.536 |
| | 12 | 3.00 | 2.00 | 3.00 | 2.00 | 0.591 |
| | 24 | 3.00 | 1.50 | 3.00 | 2.00 | 0.893 |
| | 52 | 3.00 | 2.00 | 4.00 | 2.50 | 0.555 |
| | 104 | 4.00 | 2.00 | 4.00 | 2.50 | 0.959 |
| QDASH | 0 | 53.41 | 14.82 | 52.27 | 13.64 | 0.210 |
| | 2 | 27.27 | 14.77 | 42.05 | 15.91 | 0.104 |
| | 4 | 34.09 | 13.64 | 36.36 | 14.77 | 0.295 |
| | 8 | 34.09 | 15.91 | 32.95 | 19.32 | 0.781 |
| | 12 | 26.14 | 19.32 | 29.55 | 17.05 | 0.400 |
| | 24 | 31.82 | 26.14 | 25.00 | 20.45 | 0.955 |
| | 52 | 29.55 | 19.32 | 15.91 | 24.43 | 0.525 |
| | 104 | 15.91 | 21.59 | 13.64 | 18.18 | 0.593 |
| ΔQDASH (vs week 0) | 2 | 6.82 | 10.23 | 6.81 | 14.77 | 0.701 |
| | 4 | 11.36 | 12.46 | 13.63 | 14.77 | 0.730 |
| | 8 | 12.50 | 15.92 | 15.91 | 18.30 | 0.384 |
| | 12 | 25.00 | 18.18 | 18.18 | 15.91 | 0.980 |
| | 24 | 15.91 | 15.78 | 22.50 | 20.46 | 0.347 |
| | 52 | 15.90 | 13.64 | 27.27 | 19.32 | 0.162 |
| | 104 | 27.27 | 19.31 | 34.09 | 21.59 | 0.114 |
| PRTEE | 0 | 52.25 | 12.00 | 52.25 | 14.75 | 0.550 |
| | 2 | 20.50 | 14.25 | 31.50 | 17.25 | 0.115 |
| | 4 | 23.00 | 15.00 | 25.25 | 13.50 | 0.496 |
| | 8 | 22.50 | 12.00 | 22.00 | 16.00 | 0.848 |
| | 12 | 21.25 | 14.25 | 19.75 | 15.00 | 0.920 |
| | 24 | 19.00 | 21.75 | 14.50 | 16.75 | 0.773 |
| | 52 | 18.50 | 16.50 | 11.25 | 14.63 | 0.312 |
| | 104 | 15.00 | 14.00 | 7.00 | 10.50 | 0.356 |
| ΔPRTEE (vs week 0) | 2 | 19.50 | 10.00 | 14.00 | 12.25 | **0.049** |
| | 4 | 21.50 | 12.00 | 21.50 | 13.50 | 0.743 |
| | 8 | 29.00 | 15.50 | 25.50 | 16.75 | 0.950 |
| | 12 | 29.25 | 12.25 | 29.00 | 16.75 | 0.838 |
| | 24 | 31.50 | 14.00 | 29.50 | 19.25 | 0.528 |
| | 52 | 31.50 | 13.25 | 33.50 | 19.25 | 0.311 |
| | 104 | 37.50 | 10.25 | 38.50 | 17.75 | 0.141 |

PROMs values in AA homozygotes and C allele carriers of the rs12461895 *TGFB1* gene polymorphism.

| **PROMs** | | **AA rs12461895** | | **AC+CC rs12461895** | | ***p*** |
|---|---|---|---|---|---|---|
| | week | median | ± QD | median | ± QD | **Mann-Whitney U test** |
| VAS | 0 | 7.00 | 1.50 | 5.00 | 1.50 | **0.005*** |
| | 2 | 4.00 | 1.50 | 4.00 | 1.50 | 0.562 |
| | 4 | 3.00 | 1.50 | 3.00 | 1.50 | 0.668 |
| | 8 | 3.00 | 2.00 | 3.00 | 2.00 | 0.987 |
| | 12 | 3.00 | 2.50 | 2.00 | 1.50 | 0.586 |
| | 24 | 2.00 | 2.50 | 2.00 | 2.00 | 0.799 |
| | 52 | 1.00 | 2.00 | 2.00 | 2.00 | 0.411 |
| | 104 | 0.50 | 1.00 | 1.00 | 1.50 | 0.459 |
| ΔVAS (vs week 0) | 2 | 2.00 | 1.50 | 1.00 | 1.50 | **0.012** |
| | 4 | 4.00 | 1.00 | 2.00 | 1.50 | **0.007*** |
| | 8 | 4.00 | 2.50 | 2.00 | 2.00 | **0.039** |
| | 12 | 4.00 | 2.00 | 2.00 | 2.00 | 0.132 |
| | 24 | 4.00 | 2.50 | 2.00 | 1.75 | **0.022** |
| | 52 | 5.00 | 2.50 | 2.00 | 2.00 | **0.018** |
| | 104 | 6.00 | 1.50 | 3.25 | 2.00 | **0.013** |
| QDASH | 0 | 47.72 | 13.64 | 52.27 | 13.64 | 0.945 |
| | 2 | 43.18 | 17.05 | 38.64 | 15.91 | 0.916 |
| | 4 | 34.09 | 15.91 | 36.36 | 14.20 | 0.946 |
| | 8 | 31.82 | 19.32 | 34.09 | 18.18 | 0.514 |
| | 12 | 27.27 | 23.86 | 29.55 | 14.77 | 0.601 |
| | 24 | 25.00 | 20.45 | 27.27 | 21.59 | 0.903 |
| | 52 | 12.50 | 21.59 | 20.45 | 23.86 | 0.515 |
| | 104 | 7.95 | 25.57 | 13.64 | 20.45 | 0.988 |
| ΔQDASH (vs week 0) | 2 | 4.54 | 13.63 | 6.81 | 13.64 | 0.922 |
| | 4 | 15.45 | 13.64 | 11.36 | 17.04 | 0.815 |
| | 8 | 11.36 | 19.32 | 15.91 | 18.19 | 0.588 |
| | 12 | 18.18 | 21.59 | 18.18 | 15.91 | 0.559 |
| | 24 | 22.27 | 17.05 | 20.45 | 21.02 | 0.852 |
| | 52 | 30.68 | 14.77 | 20.45 | 21.59 | 0.733 |
| | 104 | 29.54 | 22.16 | 29.55 | 22.73 | 0.831 |
| PRTEE | 0 | 54.00 | 13.75 | 52.00 | 13.75 | 0.514 |
| | 2 | 33.50 | 15.75 | 29.25 | 17.00 | 0.637 |
| | 4 | 25.00 | 12.75 | 24.50 | 14.50 | 0.700 |
| | 8 | 24.00 | 19.00 | 22.00 | 15.50 | 0.831 |
| | 12 | 16.00 | 22.75 | 20.00 | 14.25 | 0.785 |
| | 24 | 13.00 | 17.00 | 15.50 | 17.25 | 0.723 |
| | 52 | 7.75 | 13.00 | 13.00 | 16.00 | 0.320 |
| | 104 | 3.25 | 12.25 | 8.00 | 12.75 | 0.410 |
| ΔPRTEE (vs week 0) | 2 | 17.50 | 14.75 | 14.50 | 11.50 | 0.824 |
| | 4 | 25.00 | 17.00 | 21.00 | 13.00 | 0.909 |
| | 8 | 28.00 | 18.00 | 25.25 | 16.25 | 0.916 |
| | 12 | 30.00 | 20.25 | 27.50 | 15.38 | 0.836 |
| | 24 | 32.50 | 15.75 | 27.75 | 19.75 | 0.292 |
| | 52 | 38.50 | 14.75 | 32.00 | 18.75 | 0.330 |
| | 104 | 38.50 | 14.75 | 38.00 | 16.75 | 0.600 |

Legend: *TGFB1*, transforming growth factor beta 1; QD, quartile deviation; VAS, visual analog scale; QDASH, quick version of disabilities of the arm, shoulder and hand score; PRTEE, patient-rated tennis elbow evaluation; PROM, patient-reported outcome measures. *statistically significant after Hochberg correction ($p \leq 0.007$).
