# Supplementary material for: Association of the TGFB1 Gene Polymorphisms with Pain Symptoms and the Effectiveness of Platelet-Rich Plasma in the Treatment of Lateral Elbow Tendinopathy: A Prospective Cohort Study
Source: Int J Mol Sci. 2025 Mar 8;26(6):2431. doi: 10.3390/ijms26062431 (PMC11942043; doi:10.3390/ijms26062431)
Supplement: Supplementary file 1 [file ijms-26-02431-s001.zip › Supplementary Table S4.pdf]

Table S4. PROMs values (median ± QD) in carriers of different genotypes of the rs4803455 polymorphism of the *TGFB1* gene (dominant/recessive model).

PROMs values in CC homozygotes and A allele carriers of the rs4803455 *TGFB1* gene polymorphism.

**PROMs**		**CC rs4803455**		**AC+ AA rs4803455**		***p***
	week	median	± QD	median	± QD	**Mann-Whitney U test**
VAS	0	6.50	1.50	5.00	1.50	**0.021**
	2	4.00	1.50	4.00	1.50	0.691
	4	3.00	1.25	3.00	1.50	0.635
	8	3.00	2.00	3.00	2.00	0.966
	12	3.00	2.25	2.00	1.50	0.598
	24	2.00	2.00	2.00	2.00	0.656
	52	1.00	2.00	2.00	2.50	0.265
	104	1.00	1.00	1.00	1.50	0.532
ΔVAS (vs week 0)	2	2.00	1.50	1.00	1.50	**0.021**
	4	3.50	1.25	2.00	1.50	**0.027**
	8	3.50	2.50	2.00	2.00	0.086
	12	4.00	2.50	2.00	2.00	0.312
	24	3.50	2.25	2.00	2.00	**0.034**
	52	5.00	3.00	2.00	2.00	**0.026**
	104	6.00	1.50	3.00	2.00	**0.038**
QDASH	0	48.86	13.98	52.27	13.07	0.966
	2	42.05	17.05	38.64	15.91	0.851
	4	35.23	13.64	36.36	14.77	0.768
	8	30.68	18.18	34.09	18.18	0.490
	12	28.41	21.59	28.41	15.91	0.497
	24	20.45	18.75	29.55	23.30	0.640
	52	13.64	21.59	20.45	23.86	0.464
	104	9.09	13.64	13.64	21.59	0.831
ΔQDASH (vs week 0)	2	6.81	10.23	6.81	13.64	0.887
	4	14.54	13.07	11.36	17.05	0.790
	8	11.36	18.75	15.91	18.19	0.606
	12	17.04	16.48	18.18	17.05	0.499
	24	24.77	13.64	20.45	21.59	0.438
	52	29.54	15.91	19.31	21.59	0.501
	104	31.81	22.73	29.55	22.73	0.845
PRTEE	0	54.50	13.50	51.75	13.50	0.411
	2	34.50	16.38	29.00	16.75	0.378
	4	25.50	12.38	24.50	15.25	0.546
	8	25.25	17.25	21.75	15.88	0.671
	12	20.25	20.38	20.00	14.38	0.660
	24	11.50	16.00	15.75	17.88	0.702
	52	11.50	10.25	13.00	16.25	0.305
	104	7.00	8.25	7.50	14.00	0.464
ΔPRTEE (vs week 0)	2	16.00	12.38	14.75	11.50	0.924
	4	25.75	14.63	21.00	13.00	0.858
	8	26.75	19.75	25.50	15.75	0.900
	12	29.75	17.63	28.00	15.00	0.831
	24	32.25	14.00	27.50	20.75	0.169
	52	38.50	15.50	30.25	18.00	0.157
	104	38.50	14.50	37.75	16.75	0.341

PROMs values in AA homozygotes and C allele carriers of the rs4803455 *TGFB1* gene polymorphism.

**PROMs**	week	**AA rs4803455** median	**AA rs4803455** ± QD	**AC+CC rs4803455** median	**AC+CC rs4803455** ± QD	***p* Mann-Whitney U test**
VAS	0	6.00	1.00	6.00	1.88	0.717
	2	3.50	0.75	4.00	1.50	0.658
	4	2.50	2.00	3.00	1.50	0.533
	8	4.00	1.75	3.00	1.75	0.307
	12	3.50	2.25	2.00	1.50	0.801
	24	4.00	2.50	2.00	2.00	0.186
	52	3.00	2.00	1.00	2.00	0.131
	104	1.50	1.50	1.00	1.50	0.666
ΔVAS (vs week 0)	2	1.00	2.50	1.00	1.50	0.425
	4	3.00	1.50	2.00	2.00	0.362
	8	2.00	1.50	3.00	2.00	0.684
	12	3.50	2.00	3.00	2.00	0.618
	24	2.00	1.50	3.00	2.00	0.413
	52	2.00	2.00	4.00	2.50	0.472
	104	4.00	2.00	4.00	2.00	0.813
QDASH	0	56.81	11.93	52.27	14.55	0.616
	2	38.64	13.07	38.64	15.91	0.408
	4	36.36	10.80	36.36	15.91	0.816
	8	40.91	11.36	30.68	19.32	0.251
	12	34.09	17.61	27.27	16.48	0.453
	24	43.18	23.30	25.00	21.02	0.140
	52	40.91	14.77	14.77	25.00	0.050
	104	26.14	27.28	12.50	19.32	0.150
ΔQDASH (vs week 0)	2	9.09	13.75	5.68	13.63	0.820
	4	10.22	20.45	12.50	14.77	0.433
	8	4.54	18.25	15.91	18.18	0.136
	12	7.95	19.39	18.18	15.91	0.344
	24	7.96	10.80	22.72	19.32	0.097
	52	11.36	10.23	27.27	19.32	**0.032**
	104	21.59	20.39	31.82	22.73	0.141
PRTEE	0	52.75	10.63	52.25	14.63	0.727
	2	28.75	11.00	29.50	17.50	0.424
	4	25.50	11.25	24.50	14.50	0.715
	8	28.50	14.75	21.75	16.00	0.366
	12	26.50	13.63	18.50	14.38	0.345
	24	31.50	22.25	13.75	16.50	0.142
	52	21.50	10.63	10.75	15.25	**0.027**
	104	19.00	13.13	7.00	11.63	0.096
ΔPRTEE (vs week 0)	2	17.75	10.25	14.50	11.75	0.299
	4	21.00	11.88	21.50	13.75	0.782
	8	16.50	17.50	27.50	15.75	0.437
	12	20.25	16.25	29.00	16.50	0.497
	24	16.75	14.63	31.50	19.25	0.072
	52	20.25	10.63	34.00	19.00	0.151
	104	34.00	10.38	38.50	17.25	0.137

Legend: *TGFB1*, transforming growth factor beta 1; QD, quartile deviation; VAS, visual analog scale; QDASH, quick version of disabilities of the arm, shoulder and hand score; PRTEE, patient-rated tennis elbow evaluation; PROM, patient-reported outcome measures. *statistically significant after Hochberg correction ($p \leq 0.007$).
