# Supplementary material for: Association of the TGFB1 Gene Polymorphisms with Pain Symptoms and the Effectiveness of Platelet-Rich Plasma in the Treatment of Lateral Elbow Tendinopathy: A Prospective Cohort Study
Source: Int J Mol Sci. 2025 Mar 8;26(6):2431. doi: 10.3390/ijms26062431 (PMC11942043; doi:10.3390/ijms26062431)
Supplement: Supplementary file 1 [file ijms-26-02431-s001.zip › Supplementary Table S5.pdf]

Table S5. PROMs values (median ± QD) in carriers of different genotypes of the rs2241717 polymorphism of the *TGFB1* gene (dominant/recessive model).

PROMs values in CC homozygotes and A allele carriers of the rs2241717 *TGFB1* gene polymorphism.

**PROMs**		**CC rs2241717**		**AC+AA rs2241717**		***p***
	week	median	± QD	median	± QD	**Mann-Whitney U test**
VAS	0	7.00	1.50	5.00	1.50	**0.016**
	2	4.00	1.50	4.00	1.50	0.606
	4	3.00	1.25	3.00	1.50	0.804
	8	3.00	2.00	3.00	2.00	0.941
	12	3.00	2.25	3.00	1.50	0.726
	24	2.00	2.50	2.00	2.00	0.624
	52	1.00	2.00	2.00	2.00	0.430
	104	1.00	1.00	1.00	1.50	0.594
ΔVAS (vs week 0)	2	2.00	1.75	1.00	1.50	**0.029**
	4	4.00	1.00	2.00	1.50	**0.013**
	8	4.00	2.50	2.00	2.00	0.069
	12	4.00	2.25	2.00	1.50	0.192
	24	3.50	2.25	2.00	1.50	**0.037**
	52	5.00	3.00	3.00	2.00	**0.042**
	104	6.00	1.50	4.00	2.00	**0.045**
QDASH	0	47.72	13.64	52.27	12.50	0.755
	2	42.05	15.91	38.64	17.05	0.984
	4	35.23	14.20	36.36	14.77	0.821
	8	30.68	18.18	34.09	18.18	0.556
	12	27.27	21.59	29.55	14.77	0.681
	24	20.45	19.89	27.27	22.73	0.763
	52	13.64	21.59	19.32	23.86	0.611
	104	9.09	25.00	13.64	17.05	0.832
ΔQDASH (vs week 0)	2	4.54	11.93	6.81	13.64	0.899
	4	14.54	13.64	11.36	17.05	0.602
	8	11.36	17.61	15.90	18.19	0.523
	12	17.04	18.75	19.32	15.91	0.480
	24	21.36	15.34	19.31	21.59	0.788
	52	29.54	14.77	22.72	21.73	0.950
	104	27.27	20.45	34.09	21.59	0.546
PRTEE	0	52.25	13.38	52.50	14.75	0.759
	2	30.50	14.63	30.25	17.75	0.732
	4	25.50	12.63	25.50	15.00	0.778
	8	25.25	16.88	22.00	15.75	0.892
	12	19.50	20.38	21.00	14.50	0.879
	24	11.50	16.50	19.00	17.75	0.504
	52	9.50	13.00	11.50	16.25	0.395
	104	5.00	10.50	7.50	12.50	0.561
ΔPRTEE (vs week 0)	2	17.50	13.00	14.50	11.50	0.917
	4	23.50	15.88	21.50	13.00	0.962
	8	26.75	17.63	25.25	16.75	0.976
	12	29.75	19.00	27.50	15.75	0.778
	24	32.25	14.50	27.75	20.75	0.269
	52	38.50	15.75	32.00	19.00	0.466
	104	38.50	15.00	38.00	16.00	0.911

PROMs values in AA homozygotes and C allele carriers of the rs2241717 *TGFB1* gene polymorphism.

**PROMs**		**AA rs2241717**		**AC+CC rs2241717**		***p***
	week	median	± QD	median	± QD	**Mann-Whitney U test**
VAS	0	6.00	1.50	6.00	2.00	0.915
	2	4.00	2.00	4.00	1.50	0.562
	4	3.00	2.00	3.00	1.50	0.546
	8	3.50	1.75	3.00	2.00	0.496
	12	3.00	1.75	3.00	2.00	0.938
	24	3.00	3.00	2.00	2.00	0.839
	52	2.00	1.75	1.00	2.00	0.717
	104	1.00	1.50	1.00	1.50	0.798
ΔVAS (vs week 0)	2	1.00	1.50	1.00	1.50	0.974
	4	2.00	1.50	2.00	2.00	0.874
	8	2.00	1.63	3.00	2.50	0.585
	12	3.00	2.00	2.50	2.00	0.638
	24	3.00	1.50	3.00	2.00	0.971
	52	3.00	2.00	4.00	2.50	0.884
	104	4.00	1.50	4.00	2.50	0.762
QDASH	0	52.27	15.93	52.27	13.64	0.146
	2	27.27	15.91	40.91	15.91	0.075
	4	31.82	15.91	36.36	13.64	0.303
	8	36.36	17.05	31.82	19.32	0.942
	12	30.68	18.18	29.55	14.77	0.770
	24	34.09	26.14	25.00	19.89	0.863
	52	26.14	20.45	15.91	25.00	0.582
	104	13.64	21.59	12.50	16.48	0.808
ΔQDASH (vs week 0)	2	6.82	11.84	6.81	14.77	0.781
	4	11.35	12.46	13.63	14.78	0.577
	8	11.36	11.34	17.04	19.43	0.181
	12	20.45	15.89	18.18	17.04	0.526
	24	13.62	13.59	22.27	20.46	0.174
	52	15.90	14.77	29.54	19.89	0.166
	104	27.27	20.45	36.36	21.59	0.160
PRTEE	0	52.25	12.25	52.50	14.75	0.573
	2	20.00	15.50	33.00	17.75	0.076
	4	23.00	17.25	26.00	14.25	0.438
	8	24.25	11.00	22.00	16.25	0.952
	12	22.25	14.25	20.00	16.50	0.735
	24	21.50	22.25	15.00	16.63	0.582
	52	16.50	16.75	11.00	15.25	0.407
	104	8.00	14.00	7.00	10.13	0.547
ΔPRTEE (vs week 0)	2	24.00	10.00	14.00	12.50	**0.038**
	4	21.50	13.75	21.50	13.75	0.727
	8	26.75	15.50	25.50	17.25	0.782
	12	27.50	14.00	29.00	17.50	0.914
	24	30.50	14.25	30.50	19.25	0.390
	52	31.50	14.00	34.50	19.25	0.352
	104	37.50	10.50	38.50	17.75	0.215

Legend: *TGFB1*, transforming growth factor beta 1; QD, quartile deviation; VAS, visual analog scale; QDASH, quick version of disabilities of the arm, shoulder and hand score; PRTEE, patient-rated tennis elbow evaluation; PROM, patient-reported outcome measures. *statistically significant after Hochberg correction ($p \leq 0.007$).
