# Supplementary material for: Association of the TGFB1 Gene Polymorphisms with Pain Symptoms and the Effectiveness of Platelet-Rich Plasma in the Treatment of Lateral Elbow Tendinopathy: A Prospective Cohort Study
Source: Int J Mol Sci. 2025 Mar 8;26(6):2431. doi: 10.3390/ijms26062431 (PMC11942043; doi:10.3390/ijms26062431)
Supplement: Supplementary file 1 [file ijms-26-02431-s001.zip › Supplementary Table S6.pdf]

Table S6. PROMs values (median ±QD) for different genotypes of the *TGFB1* gene polymorphisms in the additive model.

**PROM**	**week**	**Genotype of rs2278422**						***p* value**			
		**CC**		**CG**		**GG**					
		**Median**	**±QD**	**Median**	**±QD**	**Median**	**±QD**	**Kruskal-Wallis test**	**CC vs CG**	**CC vs GG**	**CG vs GG**
VAS	24	1.00	1.50	3.00	2.00	3.00	2.00	0.021	**0.020**	0.283	1.000
ΔVAS	24	4.00	2.00	2.00	2.00	3.00	2.00	0.012*	**0.010**	0.278	1.000
ΔQDASH	8	17.04	19.89	18.18	15.91	4.54	17.96	0.039	1.000	**0.035**	0.199
PRTEE	24	9.50	12.13	25.50	19.25	15.00	11.50	0.049	**0.045**	1.000	0.756

**PROM**	**week**	**Genotype of rs12461895**						***p* value**			
		**AA**		**AC**		**CC**					
		**Median**	**±QD**	**Median**	**±QD**	**Median**	**±QD**	**Kruskal-Wallis test**	**AA vs AC**	**AA vs CC**	**AC vs CC**
VAS	0	7.00	1.50	5.00	2.00	6.00	1.50	0.016*	**0.014**	0.155	1.000
ΔVAS	2	2.00	1.50	1.00	1.50	1.00	1.50	0.036	**0.034**	0.266	1.000
	4	4.00	1.00	2.00	1.50	2.00	1.50	0.014*	**0.011**	0.393	0.868
	104	6.00	1.50	3.00	2.00	4.00	2.00	0.036	**0.031**	0.354	1.000

**PROM**	**week**	**Genotype of rs4803455**						***p* value**			
		**AA**		**AC**		**CC**					
		**Median**	**±QD**	**Median**	**±QD**	**Median**	**±QD**	**Kruskal-Wallis test**	**AA vs AC**	**AA vs CC**	**AC vs CC**
VAS	0	6.00	1.00	5.00	1.75	6.50	1.50	0.045	1.000	1.000	**0.043**
ΔVAS	2	1.00	2.50	1.00	1.50	2.00	1.50	0.025	0.507	1.000	**0.032**
	4	3.00	1.50	2.00	1.50	3.50	1.25	0.028	0.422	1.000	**0.039**

**PROM**	**week**	**Genotype of rs2241717**						***p* value**			
		**AA**		**AC**		**CC**					
		**Median**	**±QD**	**Median**	**±QD**	**Median**	**±QD**	**Kruskal-Wallis test**	**AA vs AC**	**AA vs CC**	**AC vs CC**
VAS	0	6.00	1.50	5.00	1.50	7.00	1.50	0.044	1.000	0.414	**0.040**
ΔVAS	4	2.00	1.50	2.00	1.50	4.00	1.00	0.028	0.994	0.542	**0.024**

Legend: *TGFB1*, transforming growth factor beta 1; QD, quartile deviation; VAS, visual analog scale; QDASH, quick version of disabilities of the arm, shoulder and hand score; PRTEE, patient-rated tennis elbow evaluation. *statistically significant after Hochberg correction ($p \leq 0.016$).
